# A functional VipA-VipB interaction is required for the type VI secretion system activity of *Vibrio cholerae* O1 strain A1552

**DOI:** 10.1186/1471-2180-13-96

**Published:** 2013-05-03

**Authors:** Jeanette E Bröms, Takahiko Ishikawa, Sun N Wai, Anders Sjöstedt

**Affiliations:** 1 Department of Clinical Microbiology, Clinical Bacteriology, Umeå University, Umeå, SE-901 85, Sweden; 2 Department of Molecular Microbiology, Umeå University, Umeå, SE-901 87, Sweden; 3 Laboratory for Molecular Infection Medicine Sweden (MIMS), Umeå University, Umeå, SE-901 87, Sweden

**Keywords:** *Vibrio cholerae*, Type VI secretion, VipA, VipB, ClpV, Hcp

## Abstract

**Background:**

Many Gram-negative bacteria rely on a type VI secretion system (T6SS) to infect eukaryotic cells or to compete against other microbes. Common to these systems is the presence of two conserved proteins, in *Vibrio cholerae* denoted VipA and VipB, which have been shown to interact in many clinically relevant pathogens. In this study, mutagenesis of a defined region within the VipA protein was used to identify residues important for VipB binding in *V. cholerae* O1 strain A1552.

**Results:**

A dramatically diminished interaction was shown to correlate with a decrease in VipB stability and a loss of hemolysin co-regulated protein (Hcp) secretion and rendered the bacterium unable to compete with *Escherichia coli* in a competition assay.

**Conclusions:**

This confirms the biological relevance of the VipA-VipB interaction, which is essential for the T6SS activity of many important human pathogens*.*

## Background

The type VI secretion system (T6SS) is a recently discovered mechanism in Gram-negative bacteria that targets secreted proteins to eukaryotic as well as prokaryotic cells [1,2]. Like type III and type IV secretion systems (T3SS and T4SS), the T6SS mediates the contact-dependent translocation of effector substrates directly into the recipient cell [3]. Although the genetic contents and organization may vary, 13 core subunits of T6SSs have been recognized [4]. Two of these are highly conserved [5], and we have demonstrated that the interaction between these proteins occurs in a range of clinically important pathogens, including *Vibrio cholerae*, *Francisella tularensis*, *Salmonella enterica*, *Escherichia coli*, *Pseudomonas aeruginosa*, and *Yersinia pseudotuberculosis*[6]. Since many of these proteins could also bind to cognate partners from other bacteria, the mechanism behind complex formation appears highly conserved. Moreover, a region encompassing a putative and conserved alpha-helix present in all of the VipA homologues of the 6 aforementioned bacteria was shown to be important for binding to their cognate partner protein [6]. Even subtle amino acid substitutions within this domain were found to result in essentially null mutant phenotypes for *F. tularensis*, neutralizing its ability to escape from the phagosomes and, thus, its ability to replicate within the cytosol of infected macrophages and rendering it avirulent [6]. The VipA-binding domain of VipB proteins has been less characterized, but may reside within the N-terminus based on recent work in *Burkholderia cenocepacia.* The same region was also shown to be necessary for the T6SS activity of *B. cenocepacia*[7].

In *V. cholerae*, VipA/VipB have been shown to form filaments that structurally resemble bacteriophage T4 contractile tail sheaths and these were quickly disassembled by ClpV, an AAA+ traffic ATPase family protein [8-10]. The tubules were shown to cycle between assembly, quick contraction, disassembly, and re-assembly, suggesting that the sheath may energize the translocation of substrates by a phage tail-like contraction mechanism [8]. The importance of ClpV for secretion of hemolysin co-regulated protein (Hcp) has been demonstrated in both *V. cholerae* V52 and *P. aeruginosa*[9,11].

In most T6SSs, Hcp and valine-glycine repeat protein G (VgrG) are exported by the secretion machinery under normal laboratory cultural conditions. This is not the case for *V. cholerae* O1 strain N16961, and therefore it was suggested that the T6SS of *V. cholerae* O1 strains was functionally inactive [12]. Our recent studies showed, however, that the T6SS of *V. cholerae* O1 strains can be activated when the bacteria are grown under high osmolarity conditions, resulting in the secretion of Hcp into the culture medium [13]. In the same study, Hcp secretion was shown to require the presence of VipA [13].

Here, residues within the previously identified VipB-binding domain of VipA (aa 104–113) [6] were exchanged to alanine as a means to identify key residues important for the interaction. To determine the biological consequences of a diminished VipA-VipB interaction in *V. cholerae* O1 strain A1552, the mutants were assessed for their ability to bind to and stabilize VipB, promote secretion of Hcp, and compete against *E. coli* in a competition assay.

## Results

### Substitutions within the large α-helix of VipA negatively impacts on VipA/VipB complex formation

**Figure 1:**
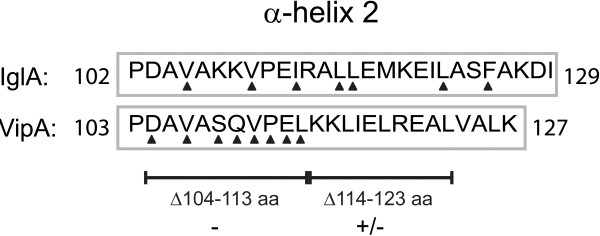
**Alanine point mutants generated within α-helix 2 of VipA.** Shown is the amino acid sequence of residues 103–127 predicted to form α-helix 2 within VipA of *V. cholerae* strain A1552 as well as the homologous region within IglA of *F. tularensis* LVS, according to Psipred (http://bioinf.cs.ucl.ac.uk/psipred/). A deletion within the first part (Δ104-113) of the α-helix abolishes VipA’s ability to bind to VipB in both B2H and Y2H systems (−), while deletions within the second part (Δ114-123) results in a VipA variant that retains VipB binding in the Y2H system, but not in the B2H system (+/−). Amino acids that were replaced with alanine in VipA are indicated by closed triangles. Residues in *F. tularensis* IglA that previously were mutated and shown to contribute to efficient IglB binding are indicated also by closed triangles [6].

**Figure 2:**
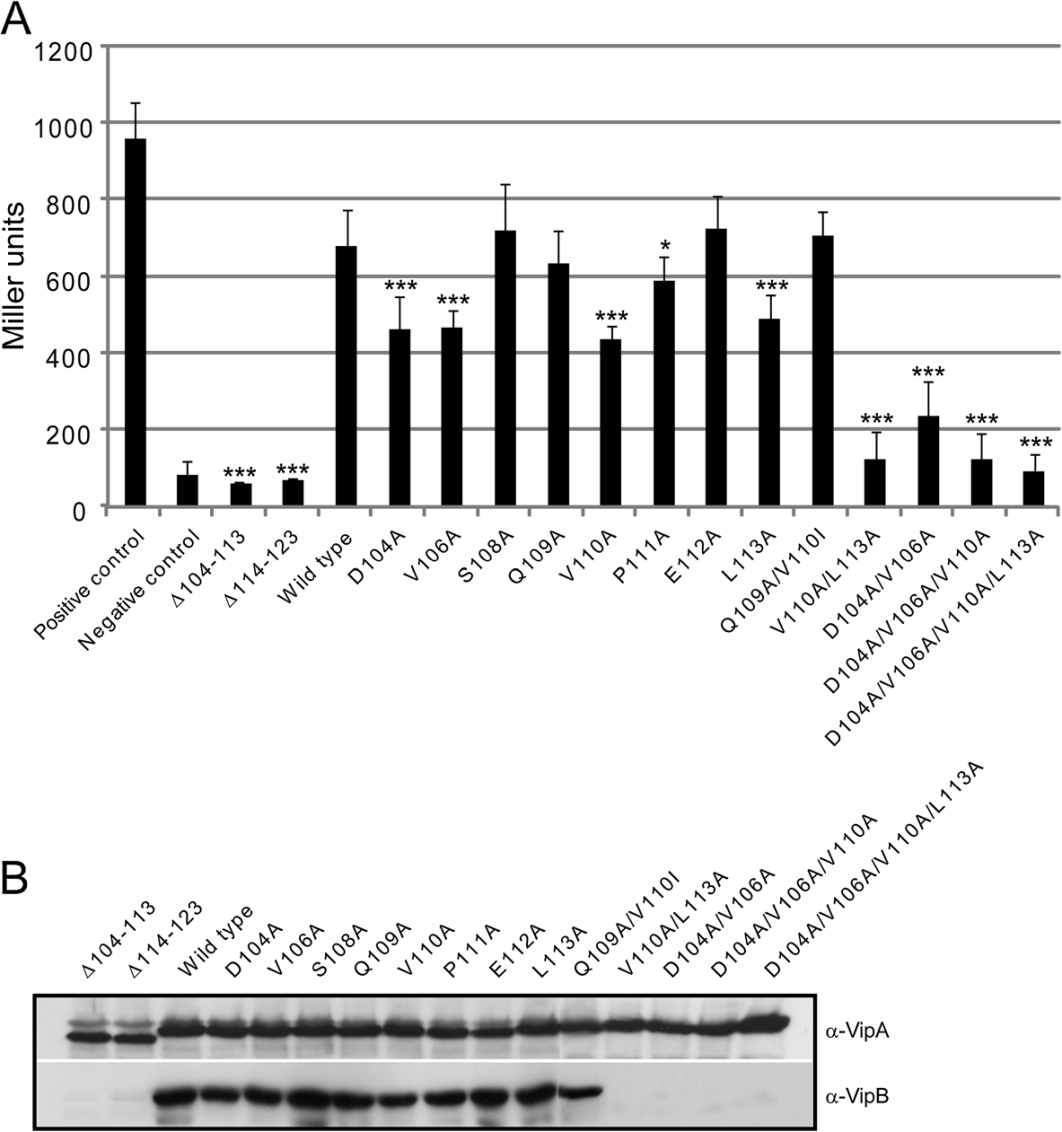
**Bacterial two-hybrid analysis of protein-protein interactions involving VipA and VipB.** (**A**) Contact between VipB and wild-type or mutant VipA, fused to Zif and to the ω subunit of *E. coli* RNAP respectively, induces transcription from the *lacZ* promoter of the *E. coli* reporter strain KDZif1ΔZ, resulting in β-galactosidase activity. As a positive control, MglA-Zif and SspA-ω was used while the negative control corresponds to empty vectors. Shown is the mean β-galactosidase activity ± standard deviation in Miller units produced from 3 independent experiments where two independent transformants were tested on each occasion. Data was subjected to a student’s 2-sided *t*-test to determine whether the β-galactosidase activity produced by a VipA mutant was significantly different from that of wild-type VipA (*, *P* < 0.05; ***, *P* < 0.001). (**B**) To determine levels of VipA mutants (upper panel) or VipB (lower panel), B2H cultures were pelleted and equivalent amounts were separated by SDS-PAGE and analyzed by Western blot using polyclonal antibodies recognizing VipA or VipB. The experiment was repeated twice.

To analyze the *V. cholerae* VipA-VipB interaction in detail, we undertook a mutagenesis-based approach. Our previous results using a yeast 2-hybrid assay (Y2H) showed that a deletion within the first part of the conserved α-helical domain of VipA (mutant Δ104-113) abolished its binding to VipB [6], while a deletion within the second part (mutant Δ114-123) did not (Bröms, unpublished) (Figure 1). To validate these results by an independent approach, we here used an *E. coli* bacterial 2-hybrid assay (B2H) for which the amount of β-galactosidase production is directly proportional to the strength of a protein-protein interaction [14]. Similar to the positive control MglA-SspA [15], VipA and VipB were found to interact efficiently in this system (Figure 2A). Deletions within the conserved α-helical domain of VipA (mutants Δ104-113 and Δ114-123) abolished its interaction to VipB in B2H (Figures 1 and 2A), suggesting that residues within region 104–123 contribute to VipB binding. To identify the key residues important for this interaction, we generated alanine substitutions, focusing on the first part of the putative α-helix (residues 104–113), since this region was shown to be crucial for VipB binding regardless of the protein-protein interaction assay used (Figure 1). Importantly, according to Psipred V2.5 (http://bioinf.cs.ucl.ac.uk/psipred/), none of the substitutions were predicted to affect the stability of the α-helix. Of the *vipA* substitution mutants generated, several were shown to exhibit small but consistent defects in VipB binding, especially mutants D104A, V106A, V110A, and L113A (Figure 2A). Importantly, V110A corresponds to the V109A substitution within *F. tularensis* IglA, which rendered *F. tularensis* unable to escape from phagosomes, grow within host cells and to cause disease in mice [6]. By combining two or more of the substitutions that had a negative impact on VipB binding, an additive effect was observed. Thus, the double mutants V110A/L113A and D104A/V106A, the triple mutant D104A/V106A/V110A and the quadruple mutant D104A/V106A/V110A/L113A were all essentially unable to bind VipB and produced β-galactosidase levels similar to the negative vector control (Figure 2A). Importantly, all VipA mutant alleles were produced at similar levels in the B2H-reporter strain KDZif1ΔZ, which rules out the possibility that variations in protein levels may account for the differences in VipB-binding (Figure 2B). VipA mutants that appeared not to bind VipB showed marked VipB instability and essentially no protein was detected by Western blot analysis (Figure 2B).

To validate the interaction data by an independent approach, we selected some of the VipA mutants and tested them for binding to VipB in the Y2H system using two independent reporter genes: *lacZ*, which allows us to compare the relative strength of the VipA-VipB interactions by quantification of β-galactosidase activity, and *MEL1*, which in the case of a positive interaction and in the presence of the substrate X-α-Gal will promote blue color development. According to both reporters, the deletion mutant Δ104-113, the double mutant V110A/L113A and the quadruple mutant D104A/V106A/V110A/L113A were all essentially unable to bind VipB and produced α-and β-galactosidase levels similar to the negative vector control, while the double mutant D104A/V106A and the triple mutant D104A/V106A/V110A both showed intermediate binding (Table 1 and data not shown). The less sensitive *MEL1* reporter assay did not detect any obvious binding defects for single mutants D104A, V106A or V110A (data not shown), while the *lacZ* reporter revealed a weak binding defect for both V106A and V110A mutants (Table 1). Thus, overall, the Y2H data confirms the results from the *E. coli* B2H assay.

**Table 1:** Protein-protein interactions in the yeast two-hybrid assay

**DNA-binding domain**	**Activation domain**	**Relative β-gal activity**
VipB	None	0.5 ± 0.1% ***
VipB	VipA	100.0 ± 5.8%
VipB	VipA _Δ104-113_	1.0 ± 0.2% ***
VipB	VipA _D104A_	92.7 ± 4.1%
VipB	VipA _V106A_	92.4 ± 3.4% *
VipB	VipA _V110A_	74.6 ± 3.4% ***
VipB	VipA _D104A/V106A_	64.1 ± 10.7% *
VipB	VipA _V110A/L113A_	1.1 ± 0.3% ***
VipB	VipA _D104A/V106A/V110A_	48.8 ± 2.0% ***
VipB	VipA _D104A/V106A/V110A/L113A_	1.0 ± 0.2% ***

VipA mutants fused to the GAL4 activation domain of plasmid pGADT7 were co-transformed with VipB on the GAL4 DNA-binding domain pGBKT7 into the *S. cerevisiae* reporter strain Y187. Activation of the *lacZ* reporter from 4 independent experiments where duplicate transformants were tested on each occasion was determined and expressed as % mean β-galactosidase activity ± SEM relative to the activity of the wild-type protein. A Student’s 2-sided *t*-test was used to determine whether the differences observed were statistically significant (*, *P* < 0.05; ***, *P* < 0.001).

**Figure 3:**
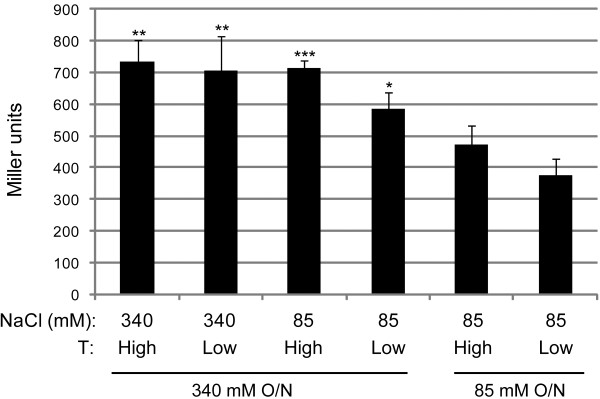
**The influence of salt and temperature on the VipA-VipB interaction.** The VipA-VipB interaction in the reporter strain KDZif1ΔZ leads to β-galactosidase activity, which is influenced by the growth temperature as well as the NaCl concentration of the medium. Shown is the mean β-galactosidase activity ± standard deviation in Miller units produced from two experiments where two independent transformants were tested on each occasion. The temperatures tested were 37°C (High) or 23°C (Low). Data was subjected to a student’s 2-sided *t*-test to determine whether the β-galactosidase activity produced at any given condition was significantly different from that produced by KDZif1ΔZ grown under standard assay conditions (85 mM NaCl, 37°C) (*, *P* < 0.05; **, *P* < 0.01; ***, *P* < 0.001).

Recently, we have shown that temperature and salinity influences the activity of the T6SS of *V. cholerae* O1 strain A1552 [13]. To determine whether salt and/or temperature also influence(s) the interaction of VipA and VipB, we compared the strength of the interaction in the B2H assay when *E. coli* was grown under different salt and temperature conditions. The results suggest that *E. coli* grown in Luria Broth (LB) supplemented with additional NaCl (high salt) over night, generally produce higher β-galactosidase activity than if grown in low salt (*i.e.* normal LB) (Figure 3). This suggests that a high concentration of salt is beneficial for the VipA-VipB interaction.

### Mutating the VipB-interaction site of VipA leads to unstable VipB and essentially abolishes Hcp secretion

**Figure 4:**
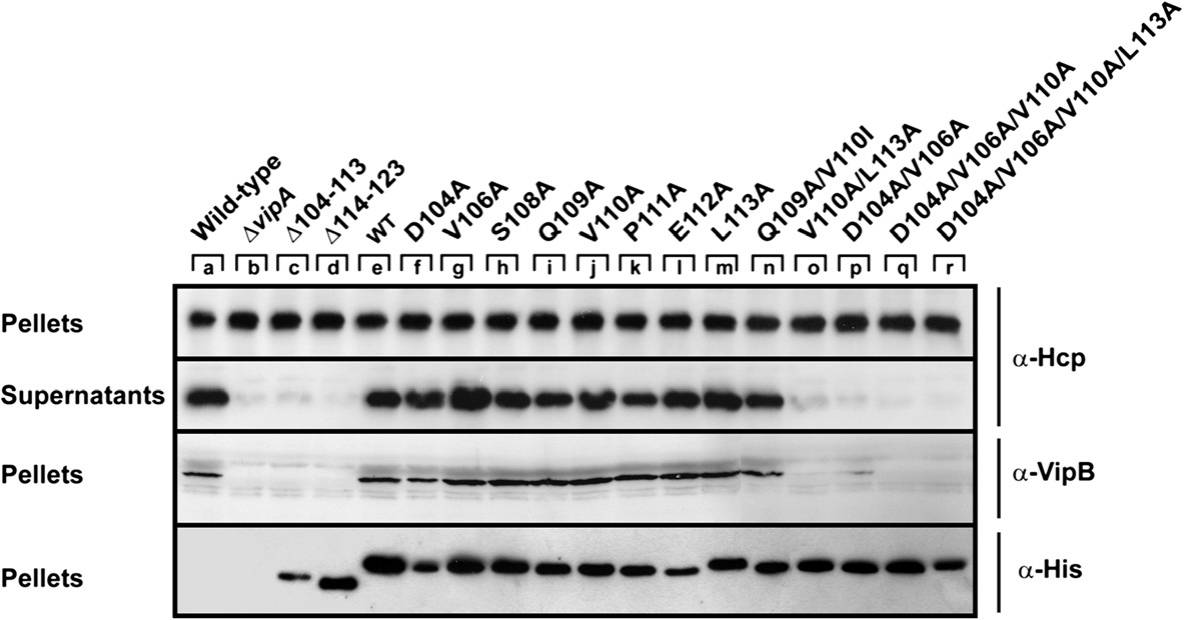
**The influence of *vipA *mutations on VipB synthesis and Hcp synthesis/secretion.** Deletion mutant alleles (lanes c-d), wild-type (lane e) or substitution mutant alleles (lanes f-r) of *vipA* were expressed from the p*tac* promoter of pMMB66EH in a *vipA* null mutant background. Hcp protein contained in the pellet fraction or secreted to the culture medium was separated by SDS-PAGE and identified by immunoblot analysis using antiserum specific for Hcp. For detection of intrabacterial VipB and VipA, anti-VipB and anti-His antisera were used respectively. The experiment was repeated at least three times and a representative example is shown.

Previously, Bönemann *et al.* have shown that VipA is essential for secretion of Hcp as well as production of VipB in *V. cholerae* non-O1 non-0139 strain V52 [9]. The latter was assumed to be a consequence of decreased VipB stability and, thereby, lower amounts of the VipA/VipB complex. We have recently shown that VipA is required for secretion of Hcp also in *V. cholerae* O1 strain A1552 [13]. To investigate if any of our *vipA* deletion or substitution mutants resulted in diminished Hcp secretion and/or VipB production, we expressed them as C-terminal His6 tagged variants from the p*tac* promoter of pMMB66EH in an A1552 *vipA* null mutant background. Importantly, His6-tagged VipA behaved identically to non-tagged VipA in all analyses performed (data not shown). By immunoblot analyses, we could confirm that all of the mutant strains expressed Hcp at levels similar to the parental strain (Figure 4, top panel), but like the *vipA* null mutant, some did not secrete Hcp into the culture medium. These corresponded to the deletion mutants Δ104-113 and Δ114-123, as well as the multiple substitution mutants V110A/L113A, D104A/V106A, D104A/V106A/V110A and D104A/V106A/V110A/L113A (Figure 4). The same mutants that failed to secrete Hcp also failed to support stable production of VipB (Figure 4), suggesting that there is a strong correlation between the ability to secrete Hcp and the ability to produce stable VipB in *V. cholerae*. When expressed together with VipB in *E. coli*, the same VipA mutants also failed to support stable VipB (compare Figures 2B and 4), demonstrating that the same mechanisms of degradation exist in these closely related species.

**Figure 5:**
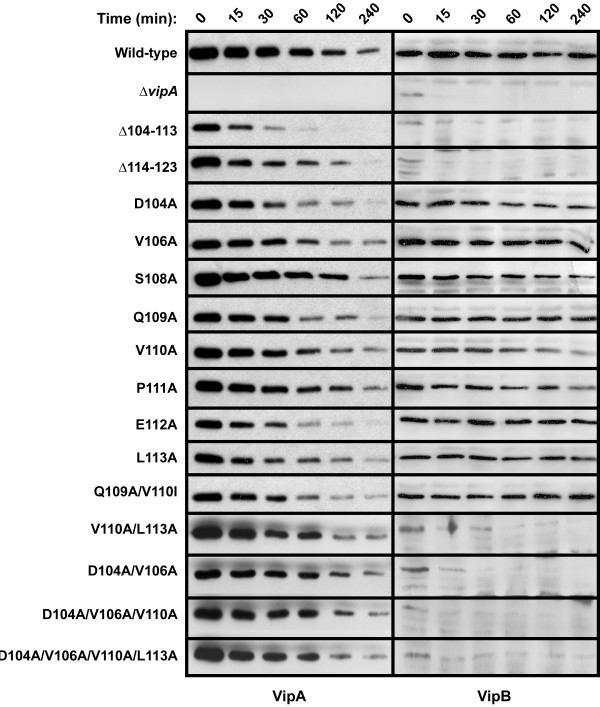
**Stability analysis of various VipA mutants and their effect on VipB stability.** Left panel: The intrabacterial stability of His6-tagged VipA mutants was examined. At time 0, chloramphenicol was added to stop new protein synthesis. Samples from pelleted bacteria were taken at different time points, and the amount of VipA protein was detected by western blot using anti-His antibodies. Right panel: The impact on VipB expression/stability exhibited by the various *vipA* mutants was investigated by western blot using anti-VipB antibodies.

Importantly, Hcp secretion as well as VipB production was efficiently restored upon expression of wild-type VipA in *trans* (Figure 4). To determine whether the drastic phenotypes of some of the mutants could be explained by a reduction in VipA stability, we used immunoblot analysis and commercially available anti-His antibodies. By this approach, reduced levels of mutants Δ104-113, D104A and E112A were consistently detected (Figure 4). Of these, only Δ104-113 exhibited a null mutant-like phenotype with respect to Hcp secretion and VipB production. No obvious reduction in the total protein levels of any of the other mutants exhibiting a null phenotype was observed (Figure 4). To further analyze the stability of the VipA mutants, we used a protein stability assay. The Δ*vipA* mutant or Δ*vipA* expressing wild-type or mutated *vipA* in *trans* were grown in LB overnight and subcultured into fresh medium supplemented with IPTG to induce VipA production. After addition of chloramphenicol to stop *de novo* protein synthesis, bacteria were collected at different time points and subjected to immunoblotting with antisera recognizing His6 (*i.e.* VipA) or VipB. In Δ*vipA* expressing wild-type VipA in *trans*, both VipA and VipB were very stable over a period of 240 min (Figure 5, top panel). In contrast, in the non-complemented Δ*vipA* mutant, VipB was barely detected in the time zero sample. We also expressed His6-tagged VipB in Δ*vipA* or Δ*vipB* mutant backgrounds and used anti-His antibodies to determine VipB stability. The overall levels of VipB were significantly lower in the Δ*vipA* strain, which was also reflected by a decrease in VipB stability over time after chloramphenicol addition (data not shown). In order to understand the effects of VipA on VipB, we also analyzed transcriptional stability of the *vipA* mutant, however, it produced *vipB* transcripts at levels similar to the parental strain A1552, -1.77 ± 0.68 (*P* = 0.17). Thus, the extreme instability of VipB in the absence of VipA is most likely due to degradation by endogenous proteases. Similar results have also been found for homologous IglA/IglB of *F. tularensis*[6]. As already observed upon analyzing the pellet samples (above), mutant Δ104-113 was significantly less stable also in the protein stability assay; it did not support VipB stability and had essentially disappeared 120 min after stopping de *novo* protein synthesis. In comparison to wild-type VipA, some of the point mutants appeared less stable over time, especially D104A and E112A, although this did not affect VipB stability (Figure 5). In contrast, none of the double, triple, or quadruple mutants appeared to be affected for VipA stability; still, VipB was very unstable in these mutant backgrounds (Figure 5). Thus, the dramatic phenotypes exhibited by some of the *vipA* substitution mutants clearly cannot be ascribed to a decrease in VipA protein stability.

### VipA/VipB complex formation influences the ability of *V. cholerae* to compete with *E. coli*

**Figure 6:**
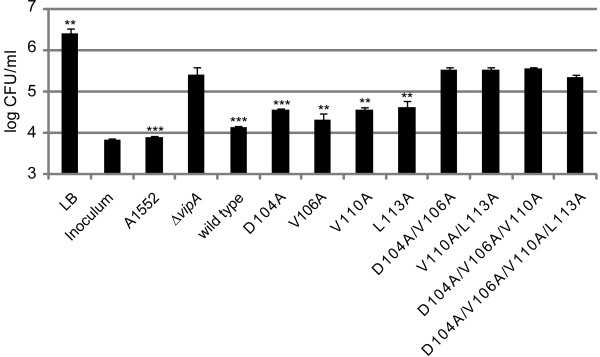
**An intact VipA-VipB interaction is important for the ability of *V. cholerae *A1552 to compete with *E. coli.****V. cholerae* parental strain A1552, Δ*vipA* and Δ*vipA* expressing wild-type VipA or mutated variants thereof were mixed (3:1) with *E. coli* MC4100 and incubated under T6SS-inducing conditions (340 mM NaCl, 37°C) on filters. After 5 h of incubation, the filters were resuspended in PBS, serially diluted and spread on *E. coli* selective plates in triplicates. Shown is the number of surviving *E. coli* (log_10_) from one representative experiment out of four. The inoculum control shows the starting number of *E. coli* prior to the 5 h incubation, while the LB control shows the number of *E. coli* obtained after 5 h of incubation in the absence of *V. cholerae*. The ability of a strain to compete with *E. coli* was compared with that of Δ*vipA* (** *P* < 0.01; *** *P* < 0.001). The experiment was repeated 4 times.

Lately, type VI secretion (T6S) has been shown to play an important role in interbacterial interactions, more specifically in bacterial killing and competition [16-20]. For example, *V. cholerae* V52 uses its T6SS to efficiently kill *E. coli*[21], which in turn requires most of the T6S genes including *vipA* and *vipB*[20]. *V. cholerae* A1552 also uses T6S to compete with *E. coli*, although it does not exert the massive T6S-mediated killing exhibited by strain V52 [13]. To investigate the ability of the A1552 *vipA* mutants to compete with *E. coli*, we used a previously established competition assay that involves mixing *V. cholerae* and *E. coli* MC4100, coculturing them on filters on agar plates at T6SS inducing conditions (*i.e.* high salt, 37°C) for 5 h, and then recovering the number of surviving target cells [13]. In addition to parental A1552 and Δ*vipA*, two categories of *vipA* mutants were used in the assay: 1) single substitution mutants D104A, V106A, V110A and L113A, which all showed slightly decreased binding to VipB, although without any obvious defects in VipB stability or Hcp secretion, and 2) multiple substitution mutants D104A/V106A, V110A/L113A, D104A/V106A/V110A and D104A/V106A/V110A/L113A, which all showed null phenotypes with respect to VipB binding, VipB stability and Hcp secretion. When *E. coli* was cocultured with parental A1552, there was a 2 log_10_ drop in the number of viable *E. coli* cells recovered compared with results for cultures inoculated with medium alone (Figure 6). However, since the numbers of viable *E. coli* never dropped below the initial inoculum, this suggests that A1552, in contrast to the highly bactericidal strain V52, may not be able to effectively kill the target cells. This may likely be explained by the observation that V52, in contrast to A1552, encodes a constitutively active T6SS that secretes high amounts of Hcp and other effector proteins [12]. Using the identical set-up, V52 was shown to efficiently kill *E. coli*, as the initial bacterial numbers dropped by > 1,000-fold (data not shown). The bacterial competition exerted by strain A1552 was shown to depend on a functional T6SS, since the number of *E. coli* increased by ~ 1.5 log_10_ when cocultured with the Δ*vipA* mutant compared to parental A1552 (Figure 6). This difference was consistent between all experiments, but was absent if *V. cholerae* was grown under non-T6S inducing conditions (LB with 85 mM NaCl) or if a Δ*hcp* mutant of A1552 was used ([13] and data not shown). By expressing wild-type *vipA* in *trans*, or any of the category 1 mutants D104A, V106A, V110A or L113A, the numbers of *E. coli* dropped to levels similar to that induced by A1552, suggesting that competition was more or less restored. Still, when compared to the wild-type protein, a small but consistent reduction in the competitive ability was observed for mutants D104A (*P* < 0.001), as well as V110A and L113A (both *P* < 0.01). In contrast, none of the multiple substitution mutants (category 2) could compete with *E. coli* and hence behaved indistinguishably from the Δ*vipA* mutant (Figure 6). Importantly, all *V. cholerae* strains tested exhibited similar growth when cultivated *in vitro* in LB (data not shown). Thus, the ability to secrete Hcp and efficiently bind/stabilize VipB is a prerequisite for the ability of A1552 to compete with *E. coli* and this in turn depends on key residues located within the conserved α-helix of VipA.

### VipA interacts with the N-terminus of ClpV in the yeast two-hybrid assay

Recently, VipA/VipB was shown to form tubular, cogwheel-like structures that are converted by a threading activity of ClpV into small complexes [9,10]. The N-domain of ClpV (residues 1–178) was shown to mediate the binding to the VipA/VipB complex, and it was suggested that the primary contact between this complex and the N-domain is mediated by VipB [9]. Recently, Pietrosiuk *et al.* identified a ClpV recognition site within VipB and showed that productive ClpV-VipB interactions require the oligomeric state of both proteins [10].

**Figure 7:**
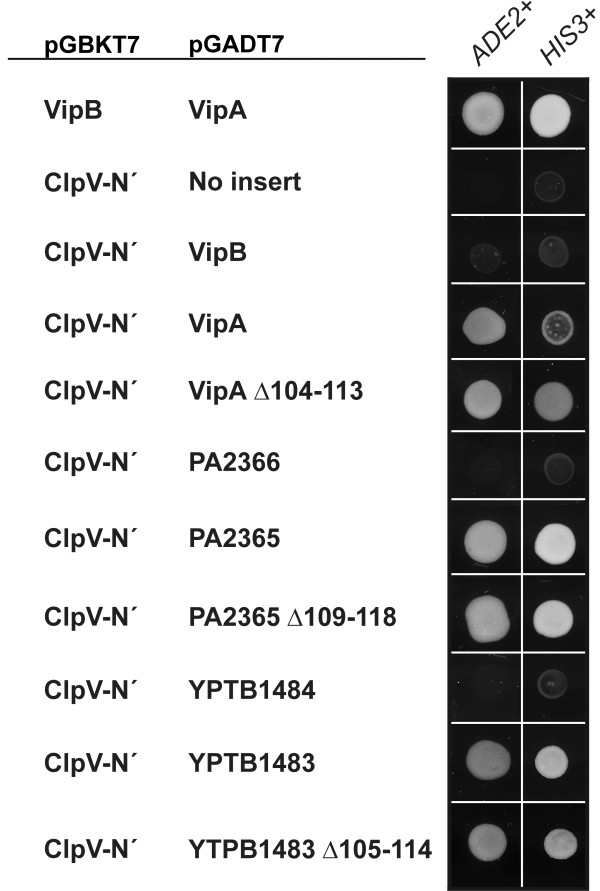
**VipA interacts with the N-terminus of ClpV (ClpV N´) in yeast.** VipA, VipB and their homologous proteins from *P. aeruginosa* PA01 (locus tag PA2365 and PA2366 respectively) or *Y. pseudotuberculosis* IP 32953 (locus tag YPTB1483 and YPTB1484 respectively) were fused to the GAL4 activation domain of plasmid pGADT7 and co-transformed with ClpV (aa 1–178) on the GAL4 DNA-binding domain pGBKT7 into the *S. cerevisiae* two-hybrid assay reporter strain AH109. A positive interaction will result in the activation of the two independent reporter genes, *ADE2* and *HIS3*, to permit growth of yeast on minimal medium devoid of adenine and histidine respectively recorded after day 5 at 25°C. Results reflect trends in growth from two independent experiments in which several individual transformants were tested on each occasion.

To study the interaction between ClpV and VipA-VipB in more detail, we used the B2H- and the Y2H systems. While B2H did not reveal any interactions between ClpV and VipA (data not shown), an interaction between VipA and the ClpV N-terminus (aa 1–178) was observed in Y2H, resulting in the activation of the reporter genes *ADE2* and *HIS3* at 25°C (Figure 7). The interaction was completely abolished at 37°C, suggesting that it was weak (data not shown). The interaction did not occur if full-length ClpV was used, which may be a consequence of the rather low expression of the latter construct (data not shown). In addition, also the VipA homologues PA2365 of *P. aeruginosa* (30% id to VipA) and YPTB1483 of *Y. pseudotuberculosis* (41% id to VipA) were shown to interact with the N-domain of *V. cholerae* ClpV in yeast, however the interaction was noticeably stronger, as it resulted in more prominent growth on medium lacking histidine (Figure 7). The ClpV interaction did not require an intact VipB-interaction site, since all of VipA Δ104-113, PA2365 Δ109-118 and YPTB1483 Δ105-114, carrying deletions within α-helix H2 [6], maintained their ClpV-interacting ability. Thus, similar to the VipA-VipB interaction, also the VipA-ClpV interaction may be conserved among T6S-containing species. Moreover, the ClpV- and VipB-interaction sites within the VipA proteins appear distinct. No interaction between ClpV and VipB or its homologues could be detected in either the B2H or the Y2H system (Figure 7 and data not shown).

## Discussion

*V. cholerae* depends on virulence factors like toxin co-regulated pili (TCP) and cholera toxin (CT), to cause the severe, life-threatening diarrheal disease, cholera [22,23]. A T6SS was recently implicated as an additional virulence determinant of *V. cholerae* that is required for Hcp secretion [12], for killing of amoeba and bacteria [12,20], and also contributes to the inflammatory diarrhea in infant mice and rabbits [24,25]. The large majority of T6SS genes (12 out of 17), including VipA, VipB, ClpV, VasF and VasK, are required for Hcp secretion, killing of amoeba and bacteria and are predicted to encode structural T6SS components [9,12,20]. In addition, regulatory proteins, VasH and VCA0122 [12,20], as well as effector proteins, VgrG-1 and possibly VCA0118, have also been identified [20,24,26,27].

By using an *in silico* approach analyzing the *F. tularensis* VipA-VipB homologues, we previously identified four distinct α-helices (H1 to H4) in the VipA homologue, IglA [6]. Interestingly, all of these helices were found to be essential for growth of *F. tularensis* in macrophages. While H3 and H4 were located in regions of little importance for VipB binding, H1 and H2 overlapped with regions crucial for the interaction. Although the *F. tularensis* T6SS is phylogenetically only distantly related to other T6SSs, domains structurally very similar to the four helices with the same specific locations were predicted in an extensive number of homologues of other Gram-negative bacteria. These structural similarities also correlated to a functional relationship, as evidenced by our demonstration of both native and heterologous interactions between the A-B homologues of 6 Gram-negative bacteria, including *Vibrio*, despite rather low levels of amino acid identities. Thus, the evidence indicates that the H2, and possibly also the H1, helices are essential for the formation of the A/B complex due to the strong preservation of these structures despite different evolutionary origins.

In view of this background, we wanted to further characterize the previously identified interaction of the H2 helix of VipA using a targeted mutagenesis approach. Residues within the conserved α-helix of VipA were exchanged to alanine and the resulting mutants tested in a B2H system. By this approach, several residues important for the VipB interaction were identified, *i.e.* D104, V106, V110, P111 and L113. Interestingly, out of these, V106, V110 and L113 were homologous to the residues V105, V109 and I112 respectively of the *F. tularensis* homologue IglA, which when mutated resulted in diminished IglB binding [6]. This confirms that the mechanism behind A/B complex formation is conserved in distantly related pathogens. The small but consistent defect in VipB-binding, however, had no visible effect on VipB expression/stability or Hcp secretion *in vitro*, although mutants D104A, V110A and L113A were all less efficient at competing with *E. coli* when tested in a bacterial competition assay. These results resemble those obtained with IglA, for which mutants V109A and L115A showed a defect in IglB binding, but not on IglB stability, yet both mutants were completely unable to grow within host cells and were also avirulent in mice [6]. Thus, even subtle defects in the A-B interaction have drastic impact on the competitive ability of T6S-containing pathogens, as well as on their ability to successfully infect host cells. By combining two or more of the single substitutions that resulted in a defect in VipB-binding, an additive effect was apparent; the ability to interact with VipB binding was poor or abolished in both B2H and Y2H systems, and similarly to a *vipA* null mutant, these multiple substitution mutants were unable to support stable VipB, Hcp secretion, and to compete with *E. coli* in a bacterial competition assay. This is the first time that this type of systematic mapping has been carried out in *Vibrio*. Importantly, the mutants provide a powerful tool for further dissecting the functional role of VipA/VipB in the *V. cholerae* T6SS.

The protein stability assay utilizing chloramphenicol to stop *de novo* protein synthesis revealed that VipB was very rapidly degraded in the absence of VipA. This indicates that VipB degradation may be a potent mechanism used by T6SS-containing bacteria to regulate the activity of the secretion system in response to distinct environmental stimuli. In further support of an important role of environmental stimuli for the VipA-VipB interaction and thereby control of T6S, we observed that a high concentration of salt appeared beneficial for the stability of the complex. High salt (340 mM) is also an important trigger for the activity of the T6SS of *V. cholerae* O1 strain A1552 [13], which is a concentration not far from that found in the normal ocean habitat of *Vibrio*, *i.e.* around 500 mM.

Overall, the results on the VipA-VipB interaction agreed between the B2H and Y2H methods. The multiple alanine substitution mutants that failed to interact with VipB, or exhibited intermediate binding, showed unstable expression of VipB in *V. cholerae* and *E. coli*, indicating a lack of proper interaction with the latter. Importantly, the failure to interact was not due to protein instability, since the mutant alleles were shown to be expressed at wild-type levels in *V. cholerae* as well as in the *E. coli* B2H system.

The exact role of the VipA/VipB complex is still elusive, but our data indicate that the functional VipA/VipB complex is a prerequisite for the normal function of the T6SS. It has been suggested to guide effector proteins to the secretion channel, analogous to what has been suggested for chaperones of type III secretion systems [28,29]. However, a study aimed to elucidate the essential function of ClpV for T6S, identified a direct interaction with VipB and revealed a remodeling of the VipA/VipB complex upon interaction with ClpV [9]. The complex alone appeared as large, tubular, cogwheel-like structures but these were dissolved when interacting with ClpV into small complexes. Moreover, no direct interaction was observed between the VipA/VipB complex and the secreted substrates Hcp or VgrG2. Thus, these findings suggest that the complex does not direct the secretory proteins for export, but instead it was proposed that the ClpV-mediated remodeling of VipA/VipB controls the dynamics of VipA/VipB tubules by regulating the number and size of the complexes and ultimately the activity of the T6S apparatus [9].

A follow-up study utilized an immobilized library of 15-mer peptides of VipA and VipB to identify the binding site between the N-terminus of ClpV and VipA/VipB [10]. While no VipA binding was identified by this approach, a few VipB peptides appeared to interact and two located in the N-terminus of VipB were subjected to further analysis. The binding was shown to involve a hydrophobic groove of ClpV, but the interaction was weak and it was hypothesized that ATP-driven ClpV hexamerization is important for coupling multiple weak interactions and to ensure a rather selective binding of ClpV to the macromolecular complex. These findings might be reconciled with those we obtained using the yeast two-hybrid interaction assay. The binding to VipB may simply be too weak to be revealed by this assay. Interestingly, the two-hybrid assay did detect binding between the N-terminus of ClpV and VipA as well as two VipA homologues encoded by *P. aeruginosa* and *Y. pseudotuberculosis*. This may be a reflection of that the peptide library used by Pietrosiuk *et al.* may not be sufficient to reveal an interaction present between the ClpV N-terminus and intact VipA proteins, since there may be secondary structures of VipA that allow its binding to ClpV. Our finding also implies that the VipA-VipB interaction with ClpV may be more complicated than previously anticipated. Although the study by Pietrosiuk *et al.* did not detect VipA degradation in a cell-free context, levels were significantly reduced when intact *V. cholerae* bacteria were analyzed, indicating that there may be direct interaction between ClpV and VipA [9].

Altogether, our findings indicate that the VipA/VipB complex has unique functional constraints and our previous findings indicate that the constraints are shared by the homologous complexes in other Gram-negative bacteria. Since VipA-VipB homologues are present in such a wide variety of pathogens, this interaction offers a unique and attractive target for the development of novel antibacterial agents. Future investigations to identify drugs that block the VipA-VipB interaction could lead to the development of therapeutics effective against a wide range of infectious diseases.

## Conclusions

VipA and VipB homologues are known to interact in many Gram-negative pathogens. In *V. cholerae*, their essential role in the secretion of T6S substrates has been demonstrated previously. Using site-directed mutagenesis within VipA, we demonstrated that a dramatically diminished interaction to VipB was shown to correlate with a decrease in VipB stability and a loss of Hcp secretion and rendered the bacterium unable to compete with *Escherichia coli* in a competition assay. This confirms the biological relevance of the VipA-VipB interaction, which is a prerequisite also for the T6S activity of intracellular pathogens like *Francisella tularensis* and *Burkholderia cenocepacia.* Thus, this conserved interaction offers an attractive target for the development of novel antibacterials.

## Methods

### Bacterial strains, plasmids and growth conditions

Bacterial strains and plasmids used in this study are listed in a table [see Additional file 1]. *E. coli* and *V. cholerae* were cultivated on Luria Bertani (LB) agar or broth at 37°C unless stated otherwise. When necessary, carbenicillin (Cb; 100 μg/ml), kanamycin (Km; 50 μg/ml), chloramphenicol (Cm; 25 μg/ml), rifampicin (Rif; 100 μg/ml), streptomycin (Strp; 50 μg/ml) or tetracycline (Tet; 10 μg/ml) were used.

### Construction of expression plasmids

Primer combinations and restriction sites used for vector construction are listed in a table [see Additional file 2]. All PCR amplified fragments were first cloned into the pCR4-TOPO TA cloning vector (Invitrogen AB) to facilitate sequencing (Eurofins MWG Operon) before proceeding with the cloning. Mutated *vipA* alleles containing in-frame deletions or codon-usage adapted alanine substitutions were constructed by overlap PCR [30]. *V. cholerae* A1552 chromosomal DNA was used as template in the PCR reactions, with the exception of the multiple substitution mutants which were constructed sequentially using previously generated substitution mutants as template. Thus, the double mutants D104A/V106A and V110A/L113A were generated using D104A and V110A respectively as template, the triple mutant D104A/V106A/V110A was generated using D104A/V106A as template and the quadruple mutant D104A/V106A/V110A/L113A was generated using D104A/V106A/ V110A as template.

For *trans*-complementation studies, PCR amplified 6 × HisC tagged *vipB* or *vipA* mutants were introduced into plasmid pMMB66EH [31] to allow expression from the p*tac* promoter and transferred into *V. cholerae* by conjugation using S17-1λ pir as donor.

To investigate protein-protein interactions in *E. coli*, PCR amplified fragments encoding VipA or mutants thereof, VipB, full-length or truncated ClpV (first 178 residues), were ligated into plasmids pBRGPω (directs the synthesis of a Gal11P-ω fusion protein and can be used to create fusions to the N-terminus of the ω subunit of *E. coli* RNAP) and pACTR-AP-Zif (directs the synthesis of the zinc finger DNA-binding domain of the murine Zif268 protein and can be used to create fusions to the N-terminus of Zif268) [32]. Plasmids were introduced into the reporter strain KDZif1ΔZ by electroporation.

To perform protein-protein interactions studies in yeast, PCR amplified fragments encoding mutant derivatives of VipA, full-length or truncated ClpV (first 178 residues), were ligated into the GAL4 activation domain plasmid pGADT7 or the GAL4 DNA-binding domain plasmid pGBKT7 (Clontech Laboratories, Palo Alto, CA, USA). To construct pGADT7 variants encoding YPTB1483 Δ105-114 and PA2365 Δ109-118, the corresponding alleles were lifted by *Nde*I/*Bam*HI and *Nde*I/*Eco*RI digestion from vectors pJEB582 and pJEB584 [6] respectively, and introduced into pGADT7. Plasmids were transferred into strain AH109 or Y187 as described previously [33].

### Analysis of T6S protein production and secretion

To induce type VI secretion in *V. cholerae* A1552 derivatives, bacterial strains were grown in LB medium containing 340 mM NaCl and samples were taken at OD_600_ = 2.0 as described previously [13]. At OD_600_ = 1.0, IPTG (Isopropyl β-D-1-thiogalactopyranoside) was added at a final concentration of 0.5 mM to induce expression from the p*tac* promoter. To assess protein secretion, TCA precipitated supernatants were analyzed, while intrabacterial protein levels were determined using total samples or pelleted bacteria. Protein samples were separated by SDS-PAGE and analyzed by Western blot using polyclonal antibodies recognizing VipB (Agrisera, Vännäs, Sweden) or Hcp [34], while VipA (His6-tagged) was visualized using monoclonal Anti-His antibodies (Qiagen, Sollentuna, Sweden). Proteins were visualized using the Enhanced Chemiluminescence system (ECL) (Amersham Biosciences, Uppsala, Sweden).

### Protein stability

The intrabacterial protein stability assay was adapted from Feldman and colleagues [35] with some modifications. In short, *V. cholerae* was grown overnight at 37°C in LB, diluted 200 × in fresh medium and grown for 1.5 h before addition of 0.5 mM IPTG. After 2 h, protein synthesis was stopped by addition of 50 μg/ml chloramphenicol (corresponds to time zero). Samples were taken out at different time points and analyzed by Western blot using antisera recognizing 6 × His or VipB (above) in combination with ECL.

### RNA extraction and qRT-PCR

RNA extraction, qRT-PCR and the sequence of the primers used have been described elsewhere [36]. For each sample, the mean cycle threshold of the test transcript was normalized to that of tmRNA [36]. Results were analysed using the delta delta Ct method of analysis and converted to relative expression ratio (2^-ΔΔCt^) for statistical analysis [37], using a paired two-tailed *t*-test to compare means. Data is presented as the mean N-fold change ± standard deviation of 2 independent experiments where triplicate samples were used.

### Bacterial two-hybrid assay (B2H)

KDZif1ΔZ reporter cells were grown overnight at 37°C in LB with appropriate antibiotics, diluted 100 × in fresh medium supplemented with antibiotics and 0.5 mM IPTG. At OD_600_ = 0.5-0.7, cells were harvested, permeabilized with SDS-CHCl_3_ and assayed for β-galactosidase activity as described [14]. To determine levels of VipA mutants or VipB, protein samples were separated by SDS-PAGE and subjected to Western blot analysis using polyclonal antibodies recognizing VipA (kind gift from Professor Axel Mogk) [9] or VipB in combination with ECL. B2H assays were performed at least three times in duplicates on separate occasions. A two-sided *t*-test with equal variance was used to determine statistical significance.

### Yeast two-hybrid assay (Y2H)

Protein expression analysis of *Saccharomyces cerevisiae* lysates and analysis of protein-protein interactions were performed according to established methods [33]. Specifically, interactions were determined by growth of yeast on synthetic dropout minimal agar (Clontech Laboratories) devoid of tryptophan, leucine (SD-LT) and adenine resulting from *ADE2* reporter gene activation. The interactive potential was confirmed by comparative growth at 25°C, 30°C and 37°C to provide an insight into the relative energy required for each interaction, and by induction of two independent reporter genes, *HIS3* and *lacZ*, by growing yeast on SD-LT agar lacking histidine and in liquid culture using ONPG (o-Nitrophenyl-beta-D-Galactopyranoside (Sigma-Aldrich, St. Louis, MO, USA) as substrate respectively. Due to an intrinsic leakiness with the *HIS3* reporter, 1.5 mM 3-aminotriazole was added to histidine dropout media to suppress false positives [38]. To monitor *MEL1* expression directly on SD-LT plates containing X-α-Gal (Sigma-Aldrich), yeast was spotted and grown for 2 days before the degree of blue colour development indicative of α-galactsidase activity and X-α-Gal hydrolysis was scored. Protein expression was verified using antibodies recognizing the activation or DNA-binding domain of GAL4 (Clontech Laboratories).

### *E. coli* competition assay

*Vibrio* and *E. coli* MC4100 (all containing empty pMMB66EH or *vipA*-expressing derivates thereof) were grown overnight at 37°C in LB medium containing 340 mM NaCl medium and Cb. Next day, strains were subcultured 1/100 in fresh medium. IPTG was added to a final concentration of 0.5 mM to *V. cholerae* strains at OD_600_ = 1.0, and upon reaching OD_600_ = 2.0, *Vibrio* was mixed at a 3 to 1 ratio with *E. coli* of OD_600_ = 0.2, followed by rigorous vortexing for 1 min. As controls, *E. coli* was also mixed with LB (LB control and inoculum control). The inoculum control, which was used to estimate the original numbers of *E. coli* in the assay, was diluted and spread immediately as described below, while 100 μL of the LB control or the *V. cholerae* - *E. coli* mixtures were incubated on 0.22 μM nitrocellulose filters (Millipore) placed on well-dried LA plates supplemented with 340 mM NaCl, Cb and IPTG. After 5 h of incubation at 37°C, bacterial cells were harvested from the filter and serial dilutions generated and spread on LA plates containing Strp (selects for *E. coli* only) in triplicates. Next day, the number of surviving *E. coli* was counted. The ability of Δ*hcp*, Δ*vipA* and Δ*vipA* expressing wild-type or mutated VipA in *trans* to compete with *E. coli* was compared.

## Abbreviations

T6S: Type VI secretion; T6SS: Type VI secretion system; T3SS: Type III secretion system; T4SS: Type IV secretion system; Hcp: Hemolysin co-regulated protein; VgrG: Valine-glycine repeat protein G; B2H: Bacterial 2-hybrid assay; Y2H: Yeast 2-hybrid assay; TCP: Toxin co-regulated pili; CT: Cholera toxin; IPTG: Isopropyl β-D-1-thiogalactopyranoside; ECL: Enhanced chemiluminescence system.

## Competing interests

The authors declare that they have no competing interests.

## Authors’ contributions

JEB generated the constructs and strains used, performed most of the analyses, contributed to the design of the study and drafted the manuscript. TI performed the qRT-PCR and contributed to the protein sample preparations and bacterial competition assays. SNW contributed to the design of the study. AS contributed to the design of the study and drafted the manuscript. All authors read and approved the final manuscript.

## Supplementary Material

Additional file 1 Strains and plasmids used in this study. Click here for file

Additional file 2 Oligonucleotides used in this study. Click here for file
