# Supplementary material for: A functional VipA-VipB interaction is required for the type VI secretion system activity of Vibrio cholerae O1 strain A1552
Source: BMC Microbiol. 2013 May 3;13:96. doi: 10.1186/1471-2180-13-96 (PMC3656785; doi:10.1186/1471-2180-13-96)
Supplement: Additional file 1 — Strains and plasmids used in this study. [file 1471-2180-13-96-S1.doc]

Table 1. Strains and plasmids used in this study

| Strain or plasmid | | Relevant genotype or phenotype | Source or reference | |
| --- | --- | --- | --- | --- |
| Strain |  | | |  |
| *E. coli* |  | | |  |
| TOP10 | F-*mcrA*, Δ(*mrr*-*hsdRMS-mcrBC*), φ80*lacZ*ΔM15, Δ*lacX*74, *recA1*, *deoR*, *araD*139, Δ(*ara-leu*)7679, *galU*, *galK*, *rpsL* (Str^R^), *endA1*, *nupG* | | | Invitrogen |
| S17-1λ*pir* | *recA*, *thi*, *pro*, *hsdR^-^M^+^*, Sm^R^, <RP4:2-Tc:Mu:Ku:Tn7>Tp^R^ | | | [^30^](#_ENREF_30) |
| MC4100 | F^-^ *araD139* Δ(*argF-lac*)*U169 rpsL150*(*strR*) *relA1 deoC1 rbsR fthD5301 fruA25λ^-^* | | | [^31^](#_ENREF_31) |
| DH5α F´IQ | F-φ80*lac*ZΔM15 Δ(*lac*ZYA-*arg*F) U169 *rec*A1 *end*A1 *hsd*R17 (rk-, mk+) *pho*A *sup*E44 λ- *thi*-1 *gyr*A96 *rel*A1/F´ *pro*AB+ *lac*IqZΔM15 zzf::Tn5 [KmR]. | | | Invitrogen |
| KDZif1ΔZ | *araD(gpt-lac)5*, *rpsL (str^R^),* Δ*spoS3::cat (Cam^R^)* *[F' lacI^q^ (Z321[-61] lacZYA*) Kan^R^]* | | | [^32^](#_ENREF_32) |
| *V. cholerae* |  | | |  |
| A1552 | O1 El Tor Inaba, Rif^R^ | | | [^33^](#_ENREF_33) |
| Δ*vipA* | A1552, carrying an in-frame deletion of *vca0107* | | | [^13^](#_ENREF_13) |
| Δ*hcp* | A1552, carrying an in-frame deletion of *hcp* | | | [^13^](#_ENREF_13) |
| V52 | O37 serotype | | | [^34^](#_ENREF_34) |
| *S. cerevisiae* |  | | |  |
| AH109 | *MATa*, *trp1-901*, *leu2-3*, *112*, *ura3-52*, *his3-200*, *gal4Δ*, *gal80Δ*, *LYS2::GAL1_UAS_-GAL1_TATA_-HIS3*, *GAL2_UAS_GAL2_TATA_-ADE2*, *URA3::MEL1_UAS_-MEL1_TATA_-lacZ*, *MEL1* | | | Clontech Laboratories |
| | Y187 | *MAT*α, *trp1-901*, *leu2-3, 112* *ura3-52*, *his3-200*, *ade2-101*, *gal4*Δ, *met–*, *gal80*Δ, *MEL1***,** *URA3*::*GAL1_UAS_-GAL1_TATA_-lacZ* | Clontech Laboratories | | --- | --- | --- | | | *MAT*α, *trp1-901*, *leu2-3, 112* *ura3-52*, *his3-200*, *ade2-101*, *gal4*Δ, *met–*, *gal80*Δ, *MEL1***,** *URA3*::*GAL1_UAS_-GAL1_TATA_-lacZ* |  | | --- | --- | | | | Clontech Laboratories |
| Plasmid |  | | |  |
| pCR®4-TOPO® | TA cloning vector, Km^R^, Ap^R^ | | | Invitrogen |
| pMMB66EH | P*tac* expression vector, Ap^R^ | | | [^35^](#_ENREF_35) |
| pJEB769 | pMMB66EH encoding VipB 6His, Ap^R^ | | | This study |
| pJEB770 | pMMB66EH encoding VipA 6His, Ap^R^ | | | This study |
| pJEB771 | pMMB66EH encoding VipA _D104A_ 6His, Ap^R^ | | | This study |
| pJEB772 | pMMB66EH encoding VipA _V106A_ 6His, Ap^R^ | | | This study |
| pJEB773 | pMMB66EH encoding VipA _S108A_ 6His, Ap^R^ | | | This study |
| pJEB774 | pMMB66EH encoding VipA _Q109A_ 6His, Ap^R^ | | | This study |
| pJEB775 | pMMB66EH encoding VipA _V110A_ 6His, Ap^R^ | | | This study |
| pJEB776 | pMMB66EH encoding VipA _Q109A/V110I_ 6His, Ap^R^ | | | This study |
| pJEB777 | pMMB66EH encoding VipA _P111A_ 6His, Ap^R^ | | | This study |
| pJEB778 | pMMB66EH encoding VipA _E112A_ 6His, Ap^R^ | | | This study |
| pJEB779 | pMMB66EH encoding VipA _L113A_ 6His, Ap^R^ | | | This study |
| pJEB842 | pMMB66EH encoding VipA _D104A/V106A_ 6His, Ap^R^ | | | This study |
| pJEB843 | pMMB66EH encoding VipA _V110A/L113A_ 6His, Ap^R^ | | | This study |
| pJEB844 | pMMB66EH encoding VipA _D104A/V106A/V110A_ 6His, Ap^R^ | | | This study |
| pJEB845 | pMMB66EH encoding VipA _D104A/V106A/V110A/L113A_ 6His, Ap^R^ | | | This study |
| pJEB780 | pMMB66EH encoding VipA _Δ104-113_ 6His, Ap^R^ | | | This study |
| pJEB781 | pMMB66EH encoding VipA _Δ114-123_ 6His, Ap^R^ | | | This study |
| pGADT7 | *LEU2*, Ap^R^ | | | Clontech Laboratories |
| pJEB564 | pGADT7 encoding VipA, *LEU2*, Ap^R^ | | | [^6^](#_ENREF_6) |
| pJEB665 | pGADT7 encoding VipA _D104A_, *LEU2*, Ap^R^ | | | This study |
| pJEB666 | pGADT7 encoding VipA _V106A_, *LEU2*, Ap^R^ | | | This study |
| pJEB606 | pGADT7 encoding VipA _V110A_, *LEU2*, Ap^R^ | | | This study |
| pJEB856 | pGADT7 encoding VipA _D104A/V106A_, *LEU2*, Ap^R^ | | | This study |
| pJEB857 | pGADT7 encoding VipA _V110A/L113A_, *LEU2*, Ap^R^ | | | This study |
| pJEB858 | pGADT7 encoding VipA _D104A/V106A/V110A_, *LEU2*, Ap^R^ | | | This study |
| pJEB859 | pGADT7 encoding VipA _D104A/V106A/V110A/L113A_, *LEU2*, Ap^R^ | | | This study |
| pJEB607 | pGADT7 encoding VipA _Δ104-113_, *LEU2*, Ap^R^ | | | This study |
| pJEB566 | pGADT7 encoding VipB, *LEU2*, Ap^R^ | | | [^6^](#_ENREF_6) |
| pJEB678 | pGADT7 encoding aa 1-178 of ClpV, *LEU2*, Ap^R^ | | | This study |
| pJEB711 | pGADT7 encoding ClpV, *LEU2*, Ap^R^ | | | This study |
| pJEB538 | pGADT7 encoding YPTB1483, *LEU2*, Ap^R^ | | | [^6^](#_ENREF_6) |
| pJEB927 | pGADT7 encoding YPTB1483 Δ105-114, *LEU2*, Ap^R^ | | | This study |
| pJEB559 | pGADT7 encoding YPTB1484, *LEU2*, Ap^R^ | | | [^6^](#_ENREF_6) |
| pJEB542 | pGADT7 encoding PA2365, *LEU2*, Ap^R^ | | | [^6^](#_ENREF_6) |
| pJEB928 | pGADT7 encoding PA2365 Δ109-118, *LEU2*, Ap^R^ | | | This study |
| pJEB555 | pGADT7 encoding PA2366, *LEU2*, Ap^R^ | | | [^6^](#_ENREF_6) |
| pGBKT7 | *TRP1*, Km^R^ | | | Clontech Laboratories |
| pJEB565 | pGBKT7 encoding VipA, *TRP1*, Km^R^ | | | [^6^](#_ENREF_6) |
| pJEB567 | pGBKT7 encoding VipB, *TRP1*, Km^R^ | | | [^6^](#_ENREF_6) |
| pJEB679 | pGBKT7 encoding aa 1-178 of ClpV, *TRP1*, Km^R^ | | | This study |
| pJEB710 | pGBKT7 encoding ClpV, *TRP1*, Km^R^ | | | This study |
| pJEB582 | pGBKT7 encoding YPTB1483 Δ105-114, *TRP1*, Km^R^ | | | [^6^](#_ENREF_6) |
| pJEB584 | pGBKT7 encoding PA2365 Δ109-118, *TRP1*, Km^R^ | | | [^6^](#_ENREF_6) |
| pACTR-AP-Zif | Zif/ω Bacterial 2-Hybrid reporter vector, Tet^R^ | | | [^32^](#_ENREF_32) |
| pJEB794 | pACTR-AP-Zif encoding VipA, Tet^R^ | | | This study |
| pJEB795 | pACTR-AP-Zif encoding VipB, Tet^R^ | | | This study |
| pJEB847 | pACTR-AP-Zif encoding ClpV, Tet^R^ | | | This study |
| pJEB793 | pACTR-AP-Zif encoding aa 1-178 of ClpV, Tet^R^ | | | This study |
| pBRGPω | Zif/ω Bacterial 2-Hybrid reporter vector, Ap^R^ | | | [^32^](#_ENREF_32) |
| pJEB798 | pBRGPω encoding VipA, Ap^R^ | | | This study |
| pJEB799 | pBRGPω encoding VipB, Ap^R^ | | | This study |
| pJEB813 | pBRGPω encoding VipA _D104A_, Ap^R^ | | | This study |
| pJEB814 | pBRGPω encoding VipA _V106A_, Ap^R^ | | | This study |
| pJEB815 | pBRGPω encoding VipA _S108A_, Ap^R^ | | | This study |
| pJEB816 | pBRGPω encoding VipA _Q109A_, Ap^R^ | | | This study |
| pJEB817 | pBRGPω encoding VipA _V110A_, Ap^R^ | | | This study |
| pJEB818 | pBRGPω encoding VipA _Q109A/V110I_, Ap^R^ | | | This study |
| pJEB819 | pBRGPω encoding VipA _P111A_, Ap^R^ | | | This study |
| pJEB820 | pBRGPω encoding VipA _E112A_, Ap^R^ | | | This study |
| pJEB821 | pBRGPω encoding VipA _L113A_, Ap^R^ | | | This study |
| pJEB825 | pBRGPω encoding VipA _D104A/V106A_, Ap^R^ | | | This study |
| pJEB833 | pBRGPω encoding VipA _V110A/L113A_, Ap^R^ | | | This study |
| pJEB832 | pBRGPω encoding VipA _D104A/V106A/V110A_, Ap^R^ | | | This study |
| pJEB834 | pBRGPω encoding VipA _D104A/V106A/V110A/L113A_, Ap^R^ | | | This study |
| pJEB822 | pBRGPω encoding VipA _Δ104-113_, Ap^R^ | | | This study |
| pJEB823 | pBRGPω encoding VipA _Δ114-123_, Ap^R^ | | | This study |
| pJEB846 | pBRGPω encoding ClpV, Ap^R^ | | | This study |
| pJEB797 | pBRGPω encoding aa 1-178 of ClpV, Ap^R^ | | | This study |
