# Supplementary material for: A functional VipA-VipB interaction is required for the type VI secretion system activity of Vibrio cholerae O1 strain A1552
Source: BMC Microbiol. 2013 May 3;13:96. doi: 10.1186/1471-2180-13-96 (PMC3656785; doi:10.1186/1471-2180-13-96)
Supplement: Additional file 2 — Oligonucleotides used in this study. [file 1471-2180-13-96-S2.doc]

Table 2. Oligonucleotides used in this study

| Purpose | Oligonucleotide pair(s) |
| --- | --- |
| *Complementation* |  |
| VipA _6His_ | VCA0107_EcoRI_F: 5´-*GAA TTC* ATG TCT AAA GAA GGA AGT GTA G-3´ (*Eco*RI) and VCA0107_6His_R: 5´-*GGA TCC* TTA GTG ATG GTG ATG GTG ATG CGC TTG TGG CTC TTC TTG ACC A-3´ (*Bam*HI) |
| VipA Δ104-113 _6His_ | VCA0107_EcoRI_F (*Eco*RI) and VCA0107_104-113_b: 5´-agg agc gaa gtc ggc taa g-3´  VCA0107_104-113_c: 5´-GCC GAC TTC GCT CCT AAA AAA TTG ATT GAG TTG CGT GAA G-3´ and VCA0107_6His_R (*Bam*HI) |
| VipA Δ114-123 _6His_ | VCA0107_EcoRI_F (*Eco*RI) and VCA0107_114-123_b: 5´-CAG TTC TGG AAC TTG TGA TG-3´  VCA0107_114-123_c: 5´-CAA GTT CCA GAA CTG GTT GCC CTT AAA GGG CCG C-3´ and VCA0107_6His_R (*Bam*HI) |
| VipA D104A _6His_ | VCA0107_EcoRI_F (*Eco*RI) and vca0107_D104a_b: 5´-A**G**C AGG AGC GAA GTC GGC TAA-3´  vca0107_D104a_c: 5´-GAC TTC GCT CCT G**C**T GCG GTG GCA TCA CAA GTT-3´ and VCA0107_6His_R (*Bam*HI) |
| VipA V106A _6His_ | VCA0107_EcoRI_F (*Eco*RI) and vca0107_V106A_b: 5´-C**G**C CGC ATC AGG AGC GAA GT-3´  vca0107_V106A_c: 5´-GCT CCT GAT GCG G**C**G GCA TCA CAA GTT CCA GAA C-3´ and VCA0107_6His_R (*Bam*HI) |
| VipA S108A _6His_ | VCA0107_EcoRI_F (*Eco*RI) and vca0107_S108A_b: 5´-TG**C** TGC CAC CGC ATC AGG AGC-3´  vca0107_S108A_c: 5´-GAT GCG GTG GCA **G**CA CAA GTT CCA GAA CTG AAA-3´ and VCA0107_6His_R (*Bam*HI) |
| VipA Q109A _6His_ | VCA0107_EcoRI_F (*Eco*RI) and vca0107_Q109A_b: 5´-T**GC** TGA TGC CAC CGC ATC AGG-3´  vca0107_Q109A_c: 5´-GCG GTG GCA TCA **GC**A GTT CCA GAA CTG AAA AAA TTG-3´ and VCA0107_6His_R (*Bam*HI) |
| VipA V110A _6His_ | VCA0107_EcoRI_F (*Eco*RI) and vca0107_v110a_b: 5´-TGG **CG**C TTG TGA TGC CAC CGC ATC-3´  vca0107_v110a_ c: 5´-GCA TCA CAA G**CG** CCA GAA CTG AAA AAA TTG AT-3´ and VCA0107_6His_R (*Bam*HI) |
| VipA P111A _6His_ | VCA0107_EcoRI_F (*Eco*RI) and vca0107_P111A_b: 5´-TG**C** AAC TTG TGA TGC CAC CGC AT-3´  vca0107_P111A_c: 5´-GCA TCA CAA GTT **G**CA GAA CTG AAA AAA TTG ATT GA-3´ and VCA0107_6His_R (*Bam*HI) |
| VipA E112A _6His_ | VCA0107_EcoRI_F (*Eco*RI) and vca0107_E112A_b: 5´-T**G**C TGG AAC TTG TGA TGC CAC-3´  vca0107_E112A_c: 5´-TCA CAA GTT CCA G**C**A CTG AAA AAA TTG ATT GAG TTG-3´ and VCA0107_6His_R (*Bam*HI) |
| VipA L113A _6His_ | VCA0107_EcoRI_F (*Eco*RI) and vca0107_L113A_b: 5´-C**GC** TTC TGG AAC TTG TGA TGC CA-3´  vca0107_L113A_c: 5´-CAA GTT CCA GAA **GC**G AAA AAA TTG ATT GAG TTG CGT G-3´ and VCA0107_6His_R (*Bam*HI) |
| VipA D104A, V106A _6His_ | VCA0107_EcoRI_F (*Eco*RI) and D104A_V106A_b: 5´-C**G**C CGC AGC AGG AGC GAA GT-3´  D104A_V106A_c: 5´-GCT CCT GCT GCG G**C**G GCA TCA CAA GTT CCA GAA C-3´ and VCA0107_6His_R (*Bam*HI) |
| VipA D104A, V106A, V110A _6His_ | VCA0107_EcoRI_F (*Eco*RI) and D104/V106/V110_b: 5´-TGG **CG**C TTG TGA TGC CGC CGC AGC-3´  vca0107_v110a_c and VCA0107_6His_R (*Bam*HI) |
| VipA D104A, V016A, V110A, L113A _6His_ | VCA0107_EcoRI_F (*Eco*RI) and D104/V106/V110/L113A_b: 5´-C**GC** TTC TGG CGC TTG TGA TGC C-3´  D104/V106/V110/L113A_c: 5´- CAA GCG CCA GAA **GC**G AAA AAA TTG ATT GAG TTG CGT G-3´ |
| VipA V110A, L113A _6His_ | VCA0107_EcoRI_F (*Eco*RI) and D104/V106/V110/L113A_b: 5´-C**GC** TTC TGG CGC TTG TGA TGC C-3´  D104/V106/V110/L113A_c: 5´- CAA GCG CCA GAA **GC**G AAA AAA TTG ATT GAG TTG CGT G-3´ |
| VipB _6His_ | VCA0108_BamHI_F: 5´-*GGA TCC* ATG ATG TCT ACG ACT GAA AAG´ (*Bam*HI) and VCA0108_6His_R: 5´-*AAG CTT* TCA GTG ATG GTG ATG GTG ATG GGC TTG ATC AAG ACG TCC AAC T-3´ (*Hind*III) |
| *Bacterial two-hybrid interaction studies* |  |
| VipA | VCA0107_F: 5´-*CAT ATG* TCT AAA GAA GGA AGT GTA G-3´ (*Nde*I) and VCA0107_R_NotI: 5´-*GCG GCC GC* CGC TTG TGG CTC TTC TTG AC (*Not*I) |
| VipA Δ104-113 | VCA0107_F (*Nde*I) and VCA0107_104-113_b  VCA0107_104-113_c and VCA0107_R_NotI (*Not*I) |
| VipA Δ114-123 | VCA0107_F (*Nde*I) and VCA0107_114-123_b  VCA0107_114-123_c and VCA0107_R_NotI (*Not*I) |
| VipA D104A | VCA0107_F (*Nde*I) and vca0107_D104a_b  vca0107_D104a_c and VCA0107_R_NotI (*Not*I) |
| VipA V106A | VCA0107_F (*Nde*I) and vca0107_V106A  vca0107_V106A_c and VCA0107_R_NotI (*Not*I) |
| VipA S108A | VCA0107_F (*Nde*I) and vca0107_S108A_b  vca0107_S108A_c and VCA0107_R_NotI (*Not*I) |
| VipA Q109A | VCA0107_F (*Nde*I) and vca0107_Q109A_b  vca0107_Q109A_c and VCA0107_R_NotI (*Not*I) |
| VipA V110A | VCA0107_F (*Nde*I) and vca0107_v110a_b  vca0107_v110a_ c and VCA0107_R_NotI (*Not*I) |
| VipA P111A | VCA0107_F (*Nde*I) and vca0107_P111A_b  vca0107_P111A_c and VCA0107_R_NotI (*Not*I) |
| VipA E112A | VCA0107_F (*Nde*I) and vca0107_E112A_b  vca0107_E112A_c and VCA0107_R_NotI (*Not*I) |
| VipA L113A | VCA0107_F (*Nde*I) and vca0107_L113A_b  vca0107_L113A_c and VCA0107_R_NotI (*Not*I) |
| VipA V110A, L113A | VCA0107_F (*Nde*I) and D104/V106/V110/L113A_b  D104/V106/V110/L113A_c and VCA0107_R_NotI (*Not*I) |
| VipA D104A, V106A | VCA0107_F (*Nde*I) and D104A_V106A_b  D104A_V106A_c and VCA0107_R_NotI (*Not*I) |
| VipA D104A, V106A, V110A | VCA0107_F (*Nde*I) and D104/V106/V110_b  vca0107_v110a_c and VCA0107_R_NotI (*Not*I) |
| VipA D104A, V016A, V110A, L113A | VCA0107_F (*Nde*I) and D104/V106/V110/L113A_b  D104/V106/V110/L113A_c and VCA0107_R_NotI (*Not*I) |
| VipB | VCA0108_F: 5´- *CAT ATG* ATG TCT ACG ACT GAA AAG-5´ (*Nde*I) and VCA0108_R_NotI: 5´- *GCG GCC GC* GGC TTG ATC AAG ACG TCC AA-3´ (*Not*I) |
| ClpV | VCA0116_NdeI_F: 5´- *CAT ATG* ATC CGT ATT GAA TTA CCC ACT-3´ (*Nde*I) and VCA0116_NotI_R: 5´- *GCG GCC GC* CGC TAC CTC TCC GAT AAA TTC A-3´(*Not*I) |
| ClpV (aa 1-178) | VCA0116_NdeI_F (*Nde*I) and VCA0116Nterm_NotI_R: 5´-*GCG GCC GC* TGG TGT TTC TGC GGC GGC T-3´ (*Not*I) |
| *Yeast two-hybrid interaction studies* |  |
| ClpV | VCA0116_EcoRI_F: 5´-*GAA TTC* GTG ATC CGT ATT GAA TTA CC-3´ (*Eco*RI) and VCA0116full_BamHI_R: *GGA TCC* TTA CGC TAC CTC TCC GAT AA-3´ (*Bam*HI) |
| ClpV (aa 1-178) | VCA0116_EcoRI_F (*Eco*RI) and VCA0116_BamHI_R: 5´-*GGA TCC* TTA TGG TGT TTC TGC GGC GG-3´ (*Bam*HI) |

The nucleotide sequences in italics represent the incorporated *Nde*I, *Not*I, *Bam*HI, *Hind*III and *Eco*RI restriction sites used for cloning of the PCR amplified DNA fragments. Underlined sequences indicate complementary sequences in the overlap PCR primers. In primers used to generate amino acid substitutions, the nucleotides substituted are indicated in boldface. To optimize expression, these substitutions were adapted according to the codon usage preferences of *V. cholerae* O1 (http://www.kazusa.or.jp/codon).
